# Cyclo-Oxygenase (COX) Inhibitors and Cardiovascular Risk: Are Non-Steroidal Anti-Inflammatory Drugs Really Anti-Inflammatory?

**DOI:** 10.3390/ijms20174262

**Published:** 2019-08-30

**Authors:** Shanzana Khan, Karen L. Andrews, Jaye P. F. Chin-Dusting

**Affiliations:** 1 Department of Pharmacology, Monash University, Clayton, Victoria 3800, Australia; 2 Baker IDI Heart and Diabetes Institute, Melbourne, Victoria 3004, Australia

**Keywords:** cyclo-oxygenase, prostanoids, T cells, immune-mediated hypertension, adaptive immunity, vascular dysfunction, coronary disease

## Abstract

Cyclo-oxygenase (COX) inhibitors are among the most commonly used drugs in the western world for their anti-inflammatory and analgesic effects. However, they are also well-known to increase the risk of coronary events. This area is of renewed significance given alarming new evidence suggesting this effect can occur even with acute usage. This contrasts with the well-established usage of aspirin as a mainstay for cardiovascular prophylaxis, as well as overwhelming evidence that COX inhibition induces vasodilation and is protective for vascular function. Here, we present an updated review of the preclinical and clinical literature regarding the cardiotoxicity of COX inhibitors. While studies to date have focussed on the role of COX in influencing renal and vascular function, we suggest an interaction between prostanoids and T cells may be a novel factor, mediating elevated cardiovascular disease risk with NSAID use.

## 1. Introduction

**Figure 1:**
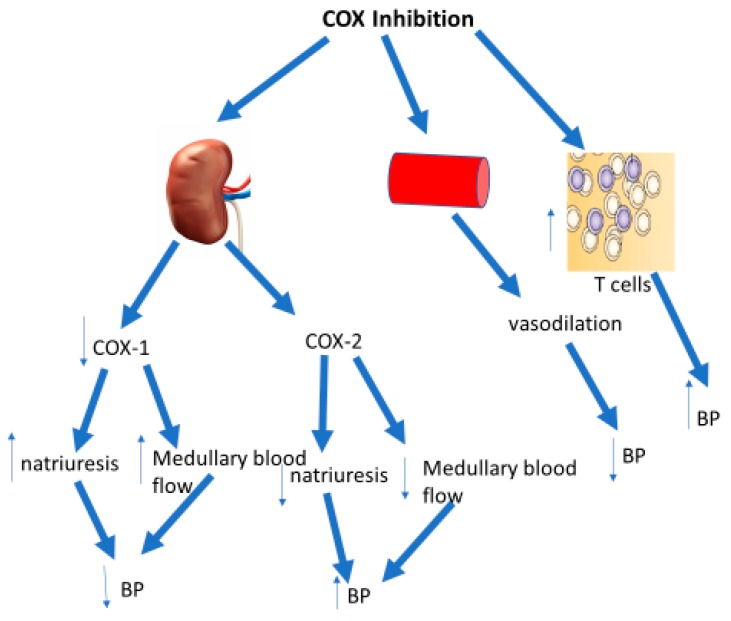
COX inhibition decreases or increases BP through inhibition of renal COX-1 or COX-2 respectively. However, the overwhelming effect of COX inhibition in the vasculature is vasodilatory. Finally, we have shown systemic COX inhibition may increase BP through activation of T cells and promotion of their infiltration into cardiovascular organs.

Cyclo-oxygenase (COX) inhibitors are among the most commonly used drugs in the world for their anti-inflammatory and analgesic properties. At least two isoforms of the enzyme exist: COX-1 and COX-2. The former, deemed the ‘constitutive’ isoform, is the dominant isoform in the body. It is ubiquitously expressed and involved in homeostatic functions, such as regulation of renal and vascular function. In contrast, COX-2 has been designated the ‘inducible’ isoform, as its basal expression is more restricted and is upregulated by inflammatory stimuli. The discovery of the COX-2 isoform in 1991 prompted the advent of selective COX-2 inhibitors to circumvent adverse gastrointestinal and renal effects associated with COX-1 inhibitors, as it was thought the COX-2 isoform was only active and expressed at sites of inflammation. However, it soon emerged that COX-2 is also constitutively expressed and regulates normal physiological functions in cardiovascular tissues, including the vasculature and the kidney and that disturbance of these housekeeping roles may have adverse cardiovascular ramifications. Interestingly, distinct opposing roles have been widely ascribed to the two COX isoforms, with COX-1 inhibition being shown to reduce BP, while COX-2 inhibition exerts a pressor effect [1] (Figure 1). The relationship between COX inhibitors and hypertension is of renewed significance, given that recent large scale clinical studies have indicated that use of NSAIDs for even one week at any dose can dramatically increase risk of MI in not only at-risk patients, but also patients with no prior history of cardiovascular disease [2]. The COX inhibitor aspirin was not included in this analysis and conversely, a concomitant intake of aspirin was considered a confounding criteria in the analysis. We have recently challenged this assumption that all doses of aspirin are cardioprotective [3], which we will discuss in this review.

We will provide an updated review on both the clinical and preclinical literature regarding COX inhibitors, hypertension, and cardiovascular disease. The effects of COX inhibitors on vascular function—an important precursor and risk factor for coronary events—will also be reviewed. We will also examine the effects of the two COX isoforms on renal function, given the well-known BP elevating effect of NSAIDs have been primarily attributed to adverse renal effects. Finally, a new dimension to the role of COX in BP regulation is added by the recent discovery that exacerbated adaptive immunity can play a fundamental role in hypertension development and disease sequelae [4]. We will thus revisit the well-established role of prostaglandins as modulators of adaptive immunity and review the evidence that prostaglandin modulation of T cell activation may represent a novel mechanism accounting for BP effects observed with use of NSAIDs and other COX inhibitors.

## 2. COX Inhibitors and Cardiovascular Disease Risk

The first evidence that COX inhibition affects cardiovascular disease (CVD) risk was uncovered by large-scale studies examining gastrointestinal outcomes as the primary endpoint. Rofecoxib and Celecoxib were the first selective COX-2 inhibitors to be marketed for their treatment of inflammatory conditions while minimising gastrointestinal disturbances attributed to COX-1 inhibition. The VIoXX Gastroinstestinal Outcomes Research Trial (VIGOR) found that although rheumatoid arthritis patients taking a fixed daily dose of Rofecoxib were less likely to experience gastrointestinal disturbances, they were five times more likely to experience MI than patients on a regimen of the non-selective COX inhibitor Naproxen [5]. The findings of this study were limited by possible cardioprotective effects of Naproxen, rendering a conclusion on the cardiotoxicity of Rofecoxib difficult without a placebo control [6,7]. Rofecoxib was withdrawn from the market in 2004 following findings of the Adenomatous Polyp PRevention On Vioxx (APPROVe) study, showing that patients taking Rofecoxib were twice as likely to experience thromboembolic events compared with a placebo treated group [8], resulting in the premature discontinuation of the trial. The increase in cardiovascular events with selective COX-2 inhibition compared with non-specific COX inhibition was hypothesised to be due to inhibition of COX-2 derived production of the vasodilator prostacyclin by the vasculature being unopposed by concomitant inhibition of COX-1 derived production of the vasoconstrictor thromboxane [9,10]. In contrast, the Celecoxib Long Term Arthritis Safety Study (CLASS) found no differences in the risk of cardiovascular events in RA and OA patients on the selective COX-2 inhibitor celecoxib compared with the non-selective COX-2 inhibitors Diclofenac and Ibuprofen [11]. These findings have been corroborated by retrospective cohort studies and other clinical studies finding no differences in CVD risk in patients on celecoxib compared with placebo [12,13,14]. Furthermore, the risk of renal events is significantly lower in osteoarthritis patients taking moderate doses of celecoxib compared with ibuprofen [15], whereas a meta-analysis of 280 trials of NSAIDs versus placebo found that high dose naproxen is associated with less risk of vascular events than other NSAIDs [16]. Thus, it appeared that CVD risk was not equal for all COX-2 inhibitors.

Questions also remained about the dose and duration of NSAID use associated with elevated CVD risk. It was suggested that the augmented cardiovascular risk with Rofecoxib use was due to the higher than standard dosage used in the VIGOR study (50 mg/day) [5,7]. Indeed, a retrospective cohort study of 378,756 individuals aged 50–85 years found that doses of Rofecoxib inferior to 25 mg did not increase risk of MI or coronary heart disease compared with celecoxib or other NSAIDs [12]. Dose was also found to be a factor in a recent large-scale nested case control study examining hospital admissions for heart failure with use of 23 traditional NSAIDs and 4 selective COX-2 inhibitors [17]. It was found that risk of admission for heart failure increased for seven traditional NSAIDs (diclofenac, ibuprofen, indomethacin, ketorolac, naproxen, nimesulide, and piroxicam) and two COX 2 inhibitors (etoricoxib and rofecoxib). This risk was doubled for diclofenac, etoricoxib, indomethacin, piroxicam, and rofecoxib used at very high doses. Although the reporting of relative rather than absolute risk may temper the severity of the conclusions of this study, alarmingly increased risk in this study was also observed in subjects with no prior history of heart failure, suggesting wider extrapolations of increased CVD risk to the general population with no predisposition to CVD.

More disconcerting evidence about the generalisability of elevated CVD risk with NSAID use was provided by Bally et al. in a large-scale meta-analysis examining the duration of NSAID use associated with increased risk of heart failure [2]. This is an important question given NSAIDs are available over the counter and their most common pattern of use is short-term relief of pain and inflammation. The authors found all traditional NSAIDs were associated with increased risk of MI, with risk again increasing with dose. Alarmingly, even 7 days of NSAID use was associated with increased risk of MI, with risk plateauing after 30 days of use. It was concluded that even intermittent ‘real world’ use of NSAIDs is associated with significantly elevated disease burden. In a follow-up nested case-control study of older adults conducted by the same group it was found that while all common NSAIDs were associated with elevated risk of MI, celecoxib required use of 30 continuous days for increased MI risk to be evident while for ibuprofen, rofecoxib, diclofenac, and naproxen heightened MI risk occurred with only 7 days of use [18]. These studies warrant a re-evaluation of NSAIDs as freely available over-the-counter products that can be acquired without consultation with health professionals.

## 3. COX Inhibitors and Hypertension Risk: Preclinical and Clinical Studies

As hypertension is the leading risk factor for cardiovascular disease (CVD), the well-described BP elevating effects of NSAIDs is likely to be a mechanism contributing to increased CVD risk with their usage. Interestingly, distinct opposing roles have been widely ascribed to the two COX isoforms in BP regulation, with inhibition of COX-1 being associated with reductions in BP in angiotensin II-induced hypertension and COX-2 inhibition being shown to elicit pressor effects [1]. This effect appears to be contingent on sodium status, as the selective COX-2 inhibitor Rofecoxib (2 mg/kg/day) increases BP in SHRSP and WKY rats on normal or high salt diets, but not on low salt diets [19]. Furthermore, Wu et al. found that rofecoxib improved cardiac hypertrophy and reduced BP in angiotensin II-infused rats on a normal sodium diet, whereas non-specific COX inhibitors ibuprofen and nimesulide were without effect [20]. Interestingly, while 6 weeks of treatment with rofecoxib and the non-specific COX inhibitor diclofenac increased BP in Dahl salt-sensitive rats, celecoxib was associated with a slight reduction in BP [21]. This is buttressed by findings by the same group that celecoxib improves vascular dysfunction in this model while rofecoxib and diclofenac are without effect [22], as well as clinical evidence that celecoxib improves endothelial function in patients with hypertension and coronary artery disease [23,24].

NSAIDs are well-documented clinically to increase BP and interfere with the efficacy of ACE-inhibitors [25]. It is speculated that the antihypertensive effects of ACE inhibitors are partially contingent on the production of vasodilatory prostaglandins, a mechanism that is not applicable to calcium-channel antagonists and diuretics. As in preclinical studies, differential effects on BP have been described depending on the NSAID used. Rofecoxib was more likely than Celecoxib to increase BP at 6 weeks in the Successive Celecoxib Efficacy Studies (SUCCESS), conducted in osteoarthritis patients aged over 65 years [26,27]. The incidence of hypertension and oedema is also higher in older osteoarthritis patients treated with rofecoxib compared with celecoxib [28]. Gastrointestinal outcome studies also support that different COX-2 inhibitors are not equal in their contribution to hypertension risk. The incidence of hypertension with rofecoxib use was 9.7% in the VIGOR study compared with 5.5% with naproxen [5] and was lower with celecoxib use compared with ibuprofen and diclofenac in the CLASS study [11]. Furthermore, it was shown in a randomised clinical trial of 178 patients with essential hypertension using 24 h ambulatory recordings that high dose celecoxib did not interfere with BP-lowering effects of the ACE-inhibitor Lisinopril over a 24 h period [29].

However, differential effects may be related to the duration of action and half-lives of various COX inhibitors. In a study of 25 black and Hispanic osteoarthritis patients, Izhar et al. examined BP increases at peak concentrations with celecoxib and diclofenac use, given the shorter half-life of the former [30]. It was found that although diclofenac increased BP more than celecoxib at 24 h, the two drugs increased BP equally at peak activity levels. Overall, systematic reviews and large-scale clinical trials comparing the effects of different NSAIDs on BP are urgently needed to reconcile differing doses, frequencies and durations of use employed by studies to date. This is critical to advise prescribing choices, whereby the choice of NSAID, dose and efficacy can be carefully balanced against potential cardiotoxicity.

## 4. COX Inhibitors and Renal Function

The pressor effects of NSAIDs have been primarily attributed to deleterious effects on renal function. It has been suggested that the two COX isoforms have opposing roles in renal function, with COX-1 inhibition enhancing natriuresis and lowering BP in angiotensin II-induced hypertension, while COX-2 inhibition elevates BP and promotes sodium retention [1]. It has also been suggested that COX-2-derived prostaglandins are predominantly vasodilators that maintain renal blood flow in the face of vasoconstrictor—such as angiotensin II and norepinephrine—with a negligible role for COX-1 derived products [1,31,32]. Indeed, elevations in renal and cortical PGE_2_ and PGI_2_ induced by angiotensin II infusion in mice is prevented through COX-2 but not COX-1 inhibition [33]. This is in contrast with previous notions that COX-1-derived prostanoids are primarily involved in the natriuretic response [7].

The two isoforms are distinctly localised in the kidney, which is thought to contribute to their polarised effects on renal function. Sodium retention with COX-2 inhibition has been primarily attributed to inhibition of medullary PGE_2_. This region is the most significant player in the regulation of NaCl and water reabsorption in the kidney. Whereas COX-1 inhibition in the mouse kidney reduces prostaglandins in renal cortex, medulla, and aorta, inhibition of COX-2 decreases prostaglandins only in renal medulla [33]. However as the expression of COX-1 in this region is far greater than COX-2, a negligible role for the latter was initially hypothesised. Inhibition of COX-1 but not COX-2 prevents natriuretic responses induced by direct renal interstitial volume expansion [34], and COX-1 (but not COX-2 inhibition) reduces sodium excretion and diuretic and natriuretic responses to furosemide in a rat model of cirrhosis and ascites [35]. It has been postulated that the role of COX-2-derived prostanoids in this region is most manifest in conditions of sodium depletion or overloading [25]. Salt loading enhances expression of COX-2 in renal medulla [36] and COX-2 inhibition reduces sodium excretion in dogs [37], in the elderly [38,39], salt-depleted human subjects [40], as well as subjects with normal renal function [11,41].

COX-2 is thought to be the predominant isoform in the macula densa (MD) of the cortex. This region is responsible for regulating renin release and afferent arteriolar tone in response to changes in luminal chloride. COX-2 inhibitors are well known to attenuate renin release in response to decreases in luminal NaCl, a phenomenon observed in vivo in a variety of disease models, in isolated perfused kidneys and in isolated perfused juxtaglomerular preparations, as reviewed by Harris et al [42]. Elevated COX-2 expression is induced by physiological conditions characterised by augmented plasma renin levels such as salt depletion, ACE inhibition or diuretic use [43,44,45,46,47]. There is also evidence of negative feedback inhibition of COX-2 expression by angiotensin and mineralocorticoids [46]. In contrast, COX-1 does not appear to play a role in regulation of renin release. In isolated juxtagomerular preparations, renin release induced by perfusion of MD with low chloride solutions is prevented by COX-2 but not COX-1 inhibition [48] and augmented renin release induced by ACE inhibitors and low salt diet was abrogated in COX-2, but not COX-1 knockout mice [49]. Inhibition of renin release with COX-2 blockade has also been shown in randomised crossover trials of healthy humans administered furosemide [50] or on a low sodium diet and COX-2 inhibition reduced hyperrinemia in patients with Bartter syndrome [51]. This effect is also likely to account for reports of altered haemodynamics, with COX-2 inhibition reducing renal blood flow in salt depleted canines and humans, but not under conditions of normal sodium intake [37,38,52]. Thus, in contrast to the sodium retaining effects of COX-2 inhibition in medulla, inhibition of this isoform suppresses renin release in the cortex. However, whether this is only observed in conditions of elevated renin release such as salt depletion remains to be clarified.

## 5. COX Inhibitors and Vascular Function

Interestingly, while the net effect of COX inhibitors comprises BP elevation, renal dysfunction, and adverse cardiovascular profiles, the dominant effects of COX inhibition on vascular function are vasoprotective. An important development in characterising endothelial control of vascular tone emerged when it was shown that acetylcholine is able to evoke contractions of vascular smooth muscle in the SHR aorta, which are prevented by COX inhibition and by endothelial denudation [53,54]. Since then it has become increasingly recognised that while basal NO levels are compromised in the SHRSP, impaired responses to acetylcholine and other endothelial stimulants are caused by increased endothelial constrictor prostanoid production, with little or no impairment in NO levels [55]. This conclusion has been primarily prompted by demonstrations that COX inhibitors restore vascular relaxation responses in the SHR to WKY levels [55]. We have confirmed the protective effects of COX inhibition in the SHRSP aorta, renal, and intrarenal arteries. The significance of constrictor prostanoids in vascular dysfunction has been corroborated by clinical studies whereby the non-selective COX inhibitor indomethacin improves acetylcholine-mediated relaxations in the forearm of essential hypertensive patients [56,57] and isolated renal arteries of aged patients [58]. However, as in renal tissue, the two isoforms of COX may play opposing roles in their influence on vascular function. While inhibition and gene knockout of COX-1 abolishes vascular dysfunction, pharmacologic, and genetic inhibition of COX-2 has minimal influence and COX-2 inhibitors have even been shown to worsen endothelial dysfunction in type 2 diabetic patients [59,60,61,62,63]. Furthermore, studies in angiotensin II-induced hypertensive mice have illustrated favourable effects of COX-1 inhibition on aortic stiffness and matrix deposition with again minimal contribution of COX-2 [64].

There is mounting evidence that the identity of the constrictor prostanoid responsible for vascular dysfunction is prostacyclin that paradoxically causes vasoconstriction via the TP receptor. Although thromboxane A_2_ is the most potent ligand of the TP receptor, other prostaglandins may activate the G-protein-coupled receptor with varying ranges of potency [65]. This is thought to be case for prostacyclin, the most abundant prostanoid in the SHR vasculature and the main product of COX-1 [66]. Although the classical action of prostacyclin is to elicit vasorelaxation and inhibit platelet aggregation through its target vasodilatory IP receptor, in the SHR, this receptor is dysfunctional before the onset of hypertension. This effect is specific to smooth muscle, as its activity is not impaired in platelets [67]. Given the production of prostacyclin is markedly higher in the SHR aorta compared with the WKY [68], IP dysfunction may be due to receptor desensitisation, although this is yet to be confirmed. Strikingly, in contrast to the WKY aorta, the peptide does not elicit vasorelaxation in the SHR aorta, but causes vasoconstriction, which is abrogated by TP receptor blockade [67]. This dysfunction is partially explained by defects in the adenylayte cyclase pathway [67]. The importance of IP functionality in cardiovascular disease is supported by findings that atherothrombosis is exacerbated in mice lacking the IP receptor [10,69] and in humans with IP receptor dysfunction [70] due to a mutation in coding genes.

The significance of TP receptors in vascular tone is underscored by reports that vascular dysfunction in a myriad of vascular beds in different species is prevented with TP receptor blockade [61,71,72,73,74,75,76]. Activation of TP receptors elicits elevated calcium and smooth muscle depolarisation through the activation of receptor-dependent and voltage-gated calcium channels, as well as Rho-kinase-induced sensitisation of myofilaments [77,78]. Although TP receptor density is similar between the WKY and SHR [60] and constrictions to synthetic agonists do not differ [59], hyperresponsiveness of the G-protein-coupled receptor to endoperoxides has been shown [59,79]. However, there are also reports that TP receptor antagonists do not improve vascular relaxation in SHR to levels seen with COX inhibitors [79], indicating the contribution of other constrictor prostanoid receptors to vascular dysfunction in the SHRSP cannot be ruled out. Apart from its vasoconstrictor and thrombotic properties, the TP receptor promotes the expression of adhesion molecules and infiltration of monocytes and macrophages [80]. Corroborating a functional antagonism between constrictor prostanoid activity and NO, TP receptor activation has been shown to inhibit NO production [81]. In vivo use of selective TP receptor antagonists improve vascular dysfunction, stiffness, and arterial remodelling in angiotensin II-induced hypertension [64] and inhibits atherogenesis in *Apoe^−/−^*-deficient mice [82]. As clinical use of COX inhibitors are associated with elevated BP [25,83], TP receptor antagonism may be envisaged as an approach to circumvent these off-target effects while capitalising on the beneficial effects of reduced constrictor prostanoid activity in the vasculature. While large scale clinical trials are pending, TP receptor antagonists have also been shown to improve endothelial function in patients with coronary artery disease, despite evoking no change in arterial BP [84].

Constrictor activity of other prostaglandins in addition to prostacyclin have also been explored. Although thromboxane A2 is the most potent ligand of the TP receptor and expression of thromboxane synthase is augmented in the SHR aorta compared with the WKY [60], others have ruled it out as the constrictor prostanoid mediating blunted responses to acetylcholine as thromboxane synthase inhibitors minimally affect vascular relaxation to the agonist [55,72,73,85]. However, this conclusion may be confounded by the inhibition of prostaglandin synthases leading to upregulation of others and diversion of endoperoxides to metabolism by other prostaglandin synthases. Furthermore, thromboxane A2 is the principal mediator of endothelium-dependent contractions caused by the calcium ionophore A23187 [86] as well as contractions to endothelin in diabetic rats [87]. Indeed, different endothelial stimulants have been shown to elicit distinct profiles of prostaglandin release, although the downstream target remains the TP [88]. These differences are speculated to be due to differences in the pattern of intracellular calcium evoked by different stimuli [89]. Prostaglandin H2 is another candidate constrictor prostanoid given it contracts the SHR aorta more potently than the WKY [59] and its production is higher in the SHR aorta, although the instability of the peptide renders it difficult to measure reliably [68]. Despite reports that prostaglandin F2α is the primary constrictor prostanoid in the hamster aorta [90], the production of prostaglandin F2α and prostaglandin E2 (PGE2) do not differ between SHR and WKY aortae [68]. Furthermore, the level of their synthases in the vasculature is markedly lower than the other mentioned prostaglandins, prompting the conclusion that their contribution to vascular dysfunction is likely to be negligible [68]. Interestingly, however, while expression of the vasoconstrictive EP3 receptor is greater in the SHR aorta than the WKY [91], it was concluded that EP receptors were not required to elicit contractions by PGE2 [91]. Conversely, the authors indicated that PGE2 acted via the TP receptor to elicit vasoconstriction. Finally, isoprotanes have been shown to exert contractile effects via the TP receptor [68], and the generation of 8-isoprostaglandin F2α production is dependent on COX activity [92]. However, mass-spectrometry studies indicate that acetylcholine does not elicit 8-isoprostaglandin F2α production [68], suggesting this can be excluded as a potential constrictor prostanoid. In all, the abundance of prostacyclin in the rat aorta, which far exceeds these other candidate prostanoids, combined with the close co-segregation of COX-1 and prostacyclin synthase, has led to the conclusion prostacyclin is the dominant constrictor prostanoid in the SHR aorta [66]. However, tyrosine nitration of prostacyclin synthase under conditions of oxidative stress may lead to upregulation of other prostanoid synthases [93], where they have been shown to play a role in impaired vascular relaxation.

## 6. COX Inhibition and Cardiac Tissue: Preclinical Studies

Several preclinical models have addressed the impact of COX inhibition on cardiac tissue and heart failure. The vasoprotective and renoprotective effects of COX-2 are paralleled by protective effects of this isoform on cardiac tissue. For example, global COX-2^−/−^ mice exhibited cardiac fibrosis compared with wildtype controls [94] and selective deletion of COX2 in cardiomyocytes resulted in reduced cardiac output and greater predisposition to arrhythmogenesis, accompanied by weight loss and reduced exercise tolerance [95]. In contrast to these findings, the COX-2 inhibitor celecoxib ameliorated cardiac hypertrophy and fibrosis induced by angiotensin II and aldosterone, while these effects were absent with rofecoxib and naproxen [96]. Furthermore, COX-2 inhibition improved left ventricular function in a murine model of doxorubicin-induced heart failure [97] and wistar rats treated with parecoxib for 5 days following coronary artery occlusion displayed improved cardiac function compared with vehicle controls [98]. This is accompanied by observations that canines treated with ASA, rofecoxib, or meclofenamate in acute MI studies show reduced infarct size [99] and that rofecoxib ameliorates cardiac fibrosis in angiotensin II induced hypertension [100]. These findings can be reconciled with deleterious effects on cardiac tissue demonstrated in COX-2 knockout mice by suggesting there may be hitherto undefined pleiotropic effects of these drugs in addition to inhibition of COX-2, with different coxibs exerting differential effects on heart failure risk, as observed in clinical studies.

It is also important to consider the downstream targets of prostanoids that could account for differential effects. Aged IP-receptor knockout mice show enhanced fibrosis compared with wildtype controls [101] and cardiac hypertrophy and fibrosis is exacerbated following banding of the aorta in IP knockout mice [102], reinforcing that the IP receptor is largely cardioprotective in heart failure. Importantly, concomitant deletion of the TP receptor rescues from cardiac fibrosis associated with IP receptor deficiency [101]. Thus, as for vascular function, the TP receptor opposes cardioprotective effects of the IP receptor. We postulate that distinct effects of pharmacological COX inhibitors may be due to differential inhibition of various prostanoids, with varying potency for the TP receptor.

Consistent with our findings, 100 mg/kg/day aspirin ameliorates cardiac hypertrophy and fibrosis in angiotensin II-induced hypertensive mice and SHR [103]. However, delivery of 120 mg/kg/day aspirin via minipump did not improve post-infarct cardiac remodelling or cardiac function after ligation of the left anterior descending coronary artery [104]. As aspirin has high oral bioavailability, the lack of clinical benefit in this study may be attributable to poor absorption via a subcutaneous route. Overall, the widespread use of aspirin as a mainstay for cardiovascular prophylaxis, along with its selectivity for COX-1, has often resulted in its inclusion as a class of its own, with less scrutiny of doses that selectively inhibit COX-1 or COX-2 as a mechanism underlying differential effects on coronary artery disease risk.

## 7. Prostaglandins and T Cells

The relationship between prostaglandins and T cells has been extensively probed in the context of autoimmune disease, as prostaglandins are the most significant lipid mediators of inflammation and homeostasis. Here, this relationship is of renewed significance in the context of hypertension pathology, given an emerging role of adaptive immunity in the aetiology of hypertension and disease sequeale, with augmented T cell infiltration into target cardiovascular organs in hypertension being shown to contribute to vascular dysfunction, sodium retention, glomerular, and cardiac fibrosis, through the production of reactive oxygen species and inflammatory cytokines [105,106]. Skewing towards inflammatory subsets Th1 and Th17 and downregulation of immunosuppressive T regulatory cell (Treg) responses and anti-inflammatory subsets has been shown to play a key role in immune-mediated hypertension [106].

The widely used anti-inflammatory effects of COX inhibitors are primarily attributed to the facilitative role of prostanoids in innate immunity. They are first-line responders to active inflammation where they promote localised vasodilation and attraction of neutrophils, macrophages and mast cells. Paradoxically however, the traditional view of the effect of prostaglandins on T cell activation has been predominantly an immunosuppressive one. The most well-characterised prostaglandin with regards to its relationship with T cells is PGE_2_, the most abundant systemic prostanoid. PGE_2_ reduces T cell proliferation and suppresses lymphomas in mice [107,108,109]. Furthermore, PGE_2_ has been shown to promote skewing of T cells towards anti-inflammatory phenotypes through inhibition of IFNɣ, but not IL-4 and IL-5 production [110], as well as to enhance induction of Tregs [111,112,113]. The production of cAMP, which interferes with the ability of IL-2 to activate T cells as well as nfĸB signalling, was found to be the primary mechanism mediating prostaglandin suppression of T cell function [114,115]. The inhibitory actions of cAMP on T cell activation are pleiotropic and act at various molecular sites of T cell activation; in addition to the mentioned pathways cAMP also abrogates T cell receptor-mediated increments in cytosolic calcium [116] and negatively regulates the phosphoinositide cycle-related transduction pathway [117,118].

The suppressive effects of PGE_2_ on T cells are also mediated via effects on antigen-presenting cells. PGE_2_ disrupts early stages of DC differentiation [119], contributing to local and systemic DC dysfunction in cancer [120,121,122] and following UV exposure [123,124]. Furthermore, migration of dendritic cells out of lymph nodes may be impeded through the induction of a tissue inhibitor of proteinase-1 by PGE_2_ [125]. PGE_2_ also enhances dendritic cell production of T cell suppressive factors such as IL-10 [119], thrombospondin-1 [126], and IDO [127], and reduces production of CCL9 [128], a chemokine that attracts naïve T cells. Dendritic cells matured in the presence of PGE_2_ display an impaired ability to elicit cytotoxic lymphocyte, Th1, and NK cell-mediated type I immunity [129,130,131], while promoting Th2 responses [130]. Suppression of Th1 polarity and cytotoxic T cells by PGE2 occurs via suppression of IL-12 production in monocytes [132] and dendritic cells [119,129] as well as IL12 receptor expression [133]. PGE_2_ is also reported to induce IL-12p40 homodimer [134,135], a competitive inhibitor of the IL-12 receptor in mice [136].

These pro-inflammatory actions of PGE_2_ are of course at variance with the potent anti-inflammatory properties of NSAIDs and the well-established therapeutic relief offered by COX inhibitors in the treatment of a vast array of autoimmune diseases driven by adaptive immune responses. A sophisticated study by Yao et al. reconciled these differences when they showed that the inhibitory property of PGE_2_ on T cell activation and Th1 responses was contingent on the level of T cell activation, whereby increasing levels of T cell-activating antibodies CD3 and CD28 in the presence of PGE_2_ overcame the initial suppressive effects of the prostanoid on T cell activation and IFNγ production [133]. Indeed, cAMP, the primary signalling molecule responsible for suppressive effects on T cell activation and Th1 responses, is known to compete with Lck activation for T cell receptor signaling [137]. Using mice deficient in each EP receptor subtype, the authors clarified that it was the EP1 and EP4 subtypes that were responsible for the Th1 promoting effect of PGE2 with strong CD28 co-stimulation and this effect was mediated via PI3K signalling instead of the cAMP pathway. Furthermore, the authors found that deletion of the prostaglandin receptors EP2 and EP4 in T cells improves colitis in vivo.

This work is accompanied by a plethora of studies that have shown prostaglandins may also promote adaptive immunity, both through priming of T cells, as well as direct effects. For example, the addition of PGE_2_ to a cocktail of inflammatory cytokines enhances dendritic cell maturation and expression of co-stimulatory molecules [138]. PGE_2_ also elicits CCR7 expression in dendritic cells [139,140], a key chemokine involved in dendritic cell migration to extralymphoid tissue, and PGE_2_-matured dendritic cells migrate to lymph nodes faster than immature dendritic cells [141]. Furthermore, dendritic cells matured in the presence of PGE_2_ exhibit an enhanced ability to promote T cell expansion [142]. PGE2 has been shown to synergise with IL-23 to promote Th17 expansion [143] and to induce expression of IL-12 in T cells to in turn promote Th1 polarisation, effects that are mediated via EP2 and EP4 receptors [133]. The impact of other prostaglandins on T cell activation and polarity is far less characterised. However PGI_2_ has been shown to act at the IP receptor to promote expression of the Th17 transcription factor RORɣt as well as IL-17A production and to decrease FOXP3 mRNA via phosphorylation of STAT3 and the cAMP-PKA pathway [144] and prostaglandin D_2_ is indispensable for the production of endothelial chemokines. Thus, while a significant role for prostaglandins in T cell activation and polarity is well-recognised, prostanoids can play a janus role depending on activation conditions, receptors and molecular pathways used.

A key question therefore remains regarding the role of prostaglandins in hypertension, a condition now recognised to have a significant autoimmune component. The clinical significance of this is further underscored by the increased prevalence of cardiovascular disease and hypertension in patients with autoimmune diseases such as rheumatoid arthritis that COX inhibitors are used to treat. COX-2 ablation was shown to induce hypertension in mice in response to a high salt diet, while wild-type mice were protected. Through elegant bone-marrow transplant experiments, Zhang et al. showed that COX-2 derived PGE2 in haematopoietic cells protects against high-salt-induced hypertension, which is associated with decreased phosphorylation of the renal sodium chloride cotransporter (NCC) [145]. This recent study is supported by previous work by Hermann et al. in which the COX-2 inhibitor rofecoxib was associated with augmented renal cytotoxic T cell infiltration in Dahl Salt Sensitive Rats as well as augmented levels of the inflammatory marker C reactive protein (CRP) [21]. Interestingly; however, the investigators found celecoxib ameliorated inflammatory renal infiltrate and CRP levels in this hypertension model, independent of an effect on BP. Again, this heterogeneity in the effects of COX-2 inhibitors remains unexplained. As previous studies by the same lab showed celecoxib to exert anti-oxidative effects [22], the reduction in renal inflammatory infiltrate observed may be due to reduced oxidative stress, as oxygen free radicals are known to recruit leukocytes through the activation of the inflammatory transcription factor nfkb which encodes for adhesion molecules [146]. Overall, it appears inhibition of the COX-2 isoform promotes adaptive immune responses in salt-sensitive hypertension. This contrasts with the efficacy of COX inhibitors in treating autoimmune conditions such as RA, lupus, colitis and contact dermatitis and their ability to reduce Th1 and Th17-mediated inflammation in these conditions. As suggested by Yao et al., perhaps different autoimmune conditions elicit differing levels of T cell activation, therefore accounting for opposing roles of COX inhibition on T cell activation [133]. Furthermore, given the effects of COX-2 inhibition are so highly dependent on sodium status, it remains to be clarified whether the suppressive effect of prostaglandins on T cells can be generalised to non-salt-sensitive hypertension.

To fill these knowledge gaps we examined the effects of chronic COX inhibition on adaptive immunity in SHRSP and WKY [3]. The COX inhibitor chosen was aspirin, given its widespread use for cardiovascular prophylaxis and because the effect of aspirin on BP is still surprisingly unclear. We chose to evaluate a dose (100 mg/kg/day) that had previously been shown to maximally inhibit both COX-1 and COX-2 [147] and had been shown to improve vascular dysfunction, cardiac hypertrophy, and oxidative stress in SHRSP and angiotensin II-induced hypertensive mice [20]. We found that 6 weeks of this high dose aspirin treatment resulted in a modest elevation in BP, but a significantly augmented adaptive immune response indicated by splenomegaly, increased circulating markers of T cell activation, and augmented renal T cell infiltration in SHRSP animals. To study the effects of aspirin on another hypertension model over a more acute timeframe, we treated mice with aspirin for two weeks with concomitant angiotensin II infusion. Similar effects of aspirin on adaptive immunity were observed in this hypertension model. In contrast to the angiotensin II-induced hypertensive model, plasma levels of angiotensin II are suppressed in the SHRSP [148], supporting that the two hypertension models are ‘opposite’ in aetiology and that the facilitative effect of COX inhibition on adaptive immunity may be applicable to hypertension of diverse aetiologies. Augmented renal T cell infiltration with aspirin was associated with renal fibrosis in both hypertension models. Interestingly, however, in agreement with our previous work, COX inhibition was protective for vascular dysfunction and cardiac fibrosis. We therefore investigated whether the cardioprotective effects of COX inhibition could be conserved with a lower dose of aspirin, while circumventing heightened adaptive immunity and BP. We chose a dose of aspirin that had previously been shown to be selective for renal COX-1 and did not affect renal COX-2 [149,150], with the hypothesis that COX-2 inhibition was responsible for adverse effects on adaptive immunity and renal fibrosis. We confirmed via gene expression studies that only 100 mg/kg/day aspirin resulted in a significant downregulation of renal COX-2. Consistent with our hypothesis, we found that 10 mg/kg/day of aspirin treatment for 6 weeks did not affect adaptive immunity while improving vascular function, cardiac and renal fibrosis in SHRSP. Our findings support that augmented adaptive immunity may be a novel mechanism underlying adverse cardiovascular profiles associated with COX-2 inhibitors, with dose being a significant factor mediating this effect.

## 8. Conclusions and Perspectives

Recent results from meta-analyses and systematic reviews have produced alarming findings that even one week of NSAID use is associated with elevated risk of MI and that this phenomenon may be applicable to those with no prior history of MI. While risk increases with increasing dosage, risk appears to plateau after one-month duration of use. Although it is well accepted that COX-2 inhibitors elevate BP through promoting sodium retention and other adverse renal consequences, similar questions addressed by these studies regarding coronary risk remain about the dose and duration of NSAID use associated with hypertension. Questions also remain about the mechanisms underlying heterogeneity of effects of different NSAIDs on BP, with evidence of pleiotropic effects which may be at play. In contrast, the effects of COX inhibition on the vasculature are overwhelmingly vasoprotective. Our understanding of the mechanisms underlying BP elevation associated with NSAIDs has been broadened by recent evidence that perturbations in adaptive immunity are a key contributor to hypertension disease sequelae, given prostaglandins are well-established modulators of adaptive immunity. Aspirin has typically been omitted in investigations regarding COX inhibitors and CVD risk, presumably because in contrast with poor cardiovascular profiles associated with NSAIDs aspirin is associated with cardioprotection and is more selective for the COX-1 isoform. Given the widely varying doses of aspirin currently used for prophylaxis and use of high dose aspirin for relief of analgesia and inflammatory conditions, we believe there is a need for clinical studies to identify the optimal dose of aspirin for cardiovascular prophylaxis, with a new understanding that higher doses may elevate BP through exacerbating adaptive immunity and renal fibrosis, while lower doses avoid these effects and harness protective effects on vascular function and cardiac fibrosis.
